# Linkage of Infection to Adverse Systemic Complications: Periodontal Disease, Toll-Like Receptors, and Other Pattern Recognition Systems

**DOI:** 10.3390/vaccines6020021

**Published:** 2018-04-05

**Authors:** Shannon M. Wallet, Vishwajeet Puri, Frank C. Gibson

**Affiliations:** 1 Department of Oral Biology, College of Dental Medicine, University of Florida, Gainesville, FL 32610, USA; swallet@dental.ufl.edu; 2 Department of Biomedical Sciences and Diabetes Institute, Ohio University, Athens, OH 45701, USA; puri@ohio.edu

**Keywords:** toll-like receptors, periodontal disease, infection, immunity, systemic disease, adipocytes, insulin resistance, lipolysis, review

## Abstract

Toll-like receptors (TLRs) are a group of pattern recognition receptors (PRRs) that provide innate immune sensing of conserved pathogen-associated molecular patterns (PAMPs) to engage early immune recognition of bacteria, viruses, and protozoa. Furthermore, TLRs provide a conduit for initiation of non-infectious inflammation following the sensing of danger-associated molecular patterns (DAMPs) generated as a consequence of cellular injury. Due to their essential role as DAMP and PAMP sensors, TLR signaling also contributes importantly to several systemic diseases including cardiovascular disease, diabetes, and others. The overlapping participation of TLRs in the control of infection, and pathogenesis of systemic diseases, has served as a starting point for research delving into the poorly defined area of infection leading to increased risk of various systemic diseases. Although conflicting studies exist, cardiovascular disease, diabetes, cancer, rheumatoid arthritis, and obesity/metabolic dysfunction have been associated with differing degrees of strength to infectious diseases. Here we will discuss elements of these connections focusing on the contributions of TLR signaling as a consequence of bacterial exposure in the context of the oral infections leading to periodontal disease, and associations with metabolic diseases including atherosclerosis and type 2 diabetes.

## 1. Introduction

In the 22 years following the seminal description in the *Drosophila* that the toll receptor is critical for controlling *Aspergillus* infection [1], and subsequent studies that humans possess an array of toll-like receptors (TLRs) that are integral for innate immunity [2,3,4], there has been an explosion of information shedding light on the molecular mechanisms that allow host innate immune sensing of foreign material [5,6]. It is now clear that an array of receptors function to recognize the conserved signatures present in these foreign molecules. Collectively, these receptors are grouped as pattern recognition receptors (PRRs) and include several families such as scavenger receptors [7,8], nucleotide binding and oligomerization domain leucine-rich repeat (LRR) containing (NLR) family (also referred to as the nucleotide binding and oligomerization domain (NOD)-like receptor family) [9,10], RIG-I-like receptors [11,12], AIM2-like receptors [13,14], receptor for advanced glycation end-products (RAGE) [15], the PYHIN (pyrin and HIN200 domain-containing) family [16], the C-type lectin receptors (CLRs) [17], the oligoadenylate synthase (OAS) proteins, and the related protein cyclic GMP-AMP (cGAMP) synthase (cGAS) [18], and toll-like receptors (TLRs) family of transmembrane receptors [19]. The innate immune system has evolved these PRRs to sense foreign molecules (both pathogenic and non-pathogenic) via recognition of what has been termed pathogenic-associated molecular patterns (PAMPs) [6]. In addition, PRRs also respond to endogenous materials released from dead and dying cells, which are referred to as danger-associated molecular patterns (DAMPs) [6]. In the context of PRR sensing, recognition of ligand initiates signaling cascades that lead to the transcription of innate immune response genes, production of products necessary to eliminate pathogens and attract immune cells, as well as to induce adaptive immune responses [20]. Thus, because of their ability to sense both PAMPs and DAMPs, PRR engagement has the potential to contribute mechanistically to inflammation-associated systemic diseases such as cardiovascular disease, cancer, rheumatoid arthritis, obesity/metabolic dysfunction, and type 2 diabetes (T2D). In this review, we will focus on the contribution of one class of PPRs, the TLRs, as a mechanistic link between bacterial infection and increased risk of atherosclerosis, metabolic disease, and T2D. Specifically, we will focus on the oral disease, periodontal disease, and illustrate its associations with these systemic inflammatory conditions using information from the bacterium *Porphyromonas gingivalis*.

## 2. Toll-Like Receptors and Innate Immune Sensing

TLRs are a collection of 13 identified receptors, 10 of which are functionally expressed in humans [5]. As a group, TLRs assemble as homo- or hetero-dimeric transmembrane receptors that recognize conserved PAMPs (and DAMPs) either extracellularly, or when partitioned in endosomal compartments [21]. Thus, thru these innate immune receptors, and the signatures they recognize, a diverse array of PAMPs from bacterial, viral, and eukaryotic pathogens that are usually detected very early during infection in a receptor-specific manner. The DAMPs that activate TLRs and other pattern-recognition receptors can be categorized into: (1) proteins secreted through a non-classical secretion mechanism involving secretory lysosomes, for example, high mobility group box (HMGB)1 and galectin-3; (2) molecules released by necrotic cells, for example, S100 proteins, HMGB1, IL-1α, galectin-3, HSP60, HSP70, HSP72, histones, and nucleic acids; and (3) extracellular matrix molecules, such as hyaluronan, heparin sulfate, fibronectin, and degraded matrix constituents. Numerous DAMPs have been identified. Theoretically, every molecule that normally resides inside cells and is extruded or is part of the extracellular matrix and is disrupted by tissue damage may potentially function as a DAMP; and hydrophobic surfaces in general have been proposed to act as DAMPs [22,23].

Following the discovery of TLR contribution to innate immune sensing of pathogen, an explosion of research ensued to identify the breadth of TLRs expressed by humans, to define the structures these receptors recognize, and to elucidate the molecular events that occur from initial ligand recognition to the development of the host immune response. Specific examples include TLR2 sensing of lipoteichoic acid, and lipopeptides [24]; TLR4 (with MD-2, LBP, and CD14) serving as a conduit of endotoxin/lipopolysaccharide (LPS) recognition [3,25], TLR5 recognition of fimbrilin [26], and TLR9 sensing of cpgDNA [27]. TLR engagement by ligand initiates a wide spectrum of responses, through a similar signaling pathway due to the presence of Toll and IL-1 receptor (TIR) domains in their cytoplasmic tails. There are 5 intracellular adaptor molecules that are recruited to the intracellular TIR domain of the TLRs to bridge signaling from the extracellular domain to the intracellular compartment [28]. The adaptor proteins Myeloid differentiation primary response 88 (MyD88) and TIR domain-contain adaptor-inducing IFN-β (TRIF) activate two independent branches of TLR signaling (MyD88-dependent and MyD88-independent), respectively. These adaptors initiate the chain of events which trigger activation and nuclear localization of transcription factors such as NF-κB and AP-1, and allow transcription of immune responsive gene controlled by these pathways [6,20,28,29].

TLR signaling is regulated at multiple levels including expression of inhibitory proteins on the cell surface that prevent ligand and/or adaptor molecule from binding to the TLR [30], or intracellularly where they inhibit or sequester signaling intermediates [31]. Regulation of TLR signaling can also be controlled by suppressor of cytokine signaling (SOCs) proteins [32], and the phosphorylation/dephosphorylation of key signaling intermediates. Inactivation of kinase activity can occur through dephosphorylation by serine-threonine phosphatases (PP2A and PP2C), tyrosine phosphatases (PTPN5, PTPN7 and PTPRR) as well as dual specificity (DUSP) phosphatases (11 exist) [31]. Several of these inhibitory molecules are induced upon TLR stimulation, thus providing feedback inhibition opportunities to terminate signaling. As such, the dynamics of TLR signaling is attributable to several factors including the type of TLR ligand(s) encountered, the concentration of these ligands, the duration of stimulation, and lastly the cell types which are engaged by these ligands [33].

Much of what has been learned regarding TLR function has centered around professional immune cells; however, TLRs are expressed and are functionally active on an array of somatic cells types such as epithelial and endothelial cells. Engagement of TLRs may result in common response between cells or can result in activation of distinctly different inflammatory pathways. For example, while both epithelial and endothelial cells express multiple TLR family members [34,35,36,37,38], there are distinct differences between the inflammatory pathways induced when compared to immune cells such as monocytes and macrophages [38]. Indeed, our group has demonstrated that while the repertoire of TLRs expressed by oral epithelial cells is similar to that expressed by macrophages, the relative amounts and ratios are significantly different, which correlated with a similar yet attenuated cytokine expression profile [37]. In addition, oral epithelial cells also had a unique response to the TLR agonist *P. gingivalis* LPS, defined by the induction of transforming growth factor (TGF)β1 and lack of interleukin (IL)-8 expression [37]. Others have demonstrated similar outcomes in endothelial cells. Specifically, in response to treatment with TLR agonists such as LPS or Pam3Cys, monocytes and macrophages robustly expressed tumor necrosis factor (TNF)-α and IL-1β, whereas endothelial cells produced little or no TNF-α or IL-1β [39,40,41], while similar levels of IL-6 and IL-8 were induced [39,42,43,44].

## 3. Periodontal Diseases and Toll-Like Receptors

Periodontal diseases, are a group of chronic inflammatory oral diseases initiated by subgingival bacterial loads that result in localized destruction of both soft and hard tissues supporting the teeth. Estimates from 2009–2012 support that approximately 64.7 million adults in the US possess clinically measurable periodontal disease, and the incidence of periodontal disease increases with advancing age [45]. Although an array of factors contributes to the risk of developing periodontal disease including oral care, environmental triggers, and host genetic make-up, it is well accepted that the a dysbiotic microbiome within the subgingival plaque initiates and sustains a chronic inflammation that is ineffective at controlling disease [46,47,48,49,50]. It is this chronic inflammation that is the principle driver of the soft and hard tissue destruction that characterizes periodontal disease.

Periodontal disease is considered a poly-microbial disease with over 700 different organisms residing in subgingival plaque [51,52]. In oral health, the subgingival biofilm is characterized by a predominating Gram-positive bacterial population; while in disease, there is a poorly understood shift in the subgingival microbiota to a predominating Gram-negative population. No one organism appears to be causally responsible for periodontal disease; rather, there are several key bacteria including *P. gingivalis* that are thought to play a pivotal role in modulating the microbial population within the subgingival plaque to a dysbiotic state that leads to this chronic inflammatory disease [53]. In addition, an array of pro-inflammatory, anti-inflammatory, and regulatory cytokines and chemokines play central roles in the soft and hard tissue destruction that characterizes periodontal disease [54,55,56,57,58,59,60]. Furthermore, the accompanying cellular infiltration observed at sites with evidence of periodontal disease is also complex, consisting of mononuclear cells, dendritic cells, B and T lymphocytes, and neutrophils. The neutrophil is best understood as an acute inflammatory responder cell with a primary role to combat infection; however, more recent evidence supports that neutrophils can interact with a variety of cells such as macrophages, B cells, and T lymphocytes [61,62,63], to help shape innate and adaptive immunity. In addition to innate immune sensing thru receptors such as TLRs, neutrophil activation can occur via several mechanisms including complement cascade components such C5a [64]. Interestingly, complement activation in some instances can be enhanced thru interactions with TLR signaling [65]. These cells provide maintenance of periodontal health while in disease, these cells produce an array of inflammatory mediators including reactive oxygen species and CCL2 and CCL20 chemokines which are necessary for recruitment of Th17 cell [66]. Individuals with leukocyte adhesion disease (LAD)-1 are particularly susceptible to periodontal disease, and experience severe forms if disease as they lack neutrophil recruitment [67], and possess dysregulated Th17 immunity that leads to elevated oral bone loss to bacterial exposure [68].

**Figure 1:**
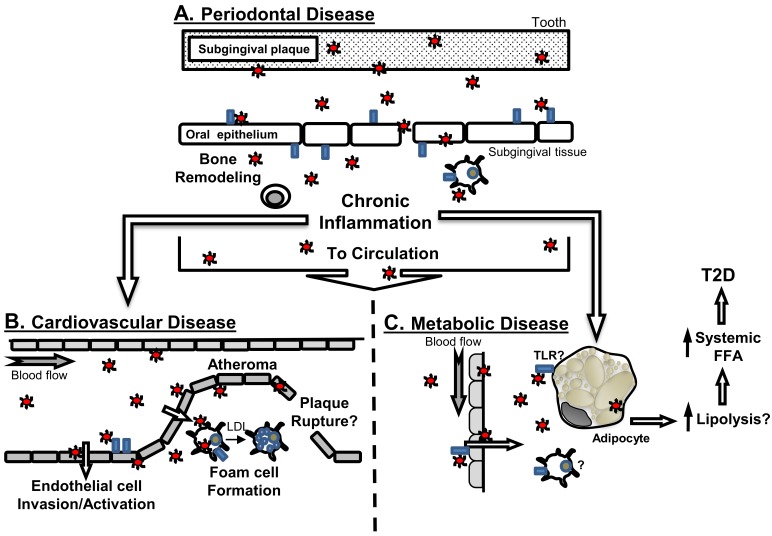
Connections of periodontal disease to systemic diseases and implication of TLRs. (**A**) Periodontal disease is an oral disease whereby bacteria such as *P. gingivalis* (red circles) interact with oral epithelial cells in part through TLRs (blue rectangles). This initial host interaction participates in the generation of chronic local inflammation, and promotes immune cell recruitment to affected gingival tissues. *P. gingivalis* and other periodontal disease-associated bacteria have been detected in blood, and thus can transition from the oral cavity via the circulation, or potentially via translocation within immune cells that could migrate from the oral environment, to localize at distant sites, and influence; (**B**) cardiovascular disease, or (**C**) metabolic disease (hypothetical model). Animal modeling supports that *P. gingivalis* can accelerate atherosclerosis, and this is in part mediated by activities of TLRs, at the vascular endothelium or as part of bacterial interaction with macrophages to promote foam cell formation. The resultant inflammation may further promote development of increasing vascular plaque complexity associated with atherosclerosis. As a consequence of bacteremia, *P. gingivalis* may gain access to adipose tissue and interact with adipocytes either directly, or indirectly via inflammatory cells and/or chronic inflammatory cues sensed by adipose tissue. Through these direct or indirect strategies, adipocytes would elevate lipolysis, that would contribute to increased systemic levels of free fatty acids (FFA) to promotes insulin resistance and ultimately lead to the development of T2D. The contribution of TLRs to oral infection-influenced adipocyte dysfunction is unknown.

*P. gingivalis* stimulates oral bone loss in part through TLR2 [69,70]. Indeed, TLR2 also plays a role in neutrophil recognition of *P. gingivalis* [71]. TLR2 signals via the adaptor molecule MyD88. Interestingly, *P. gingivalis* possess the capacity to modulate C5a signaling in neutrophils, which leads to MyD88 degradation thru a TLR2-mediated pathway (C5aR-TLR2 cross-talk) involving the ubiquitin ligase Smurf1, thus leading to reduced antimicrobial killing. In the absence of MyD88, alternative TLR2 signaling occurs via Mal and PI3kinase (PI3K) to evoke inflammatory activation characterized by elevated levels of pro-inflammatory cytokine production, with limited ability to actively take up foreign material [50]. Indeed, blocking C5aR-TLR2 cross-talk reduced production of inflammatory markers, and allowed for more effective host clearance of *P. gingivalis* following oral infection [72]. Despite these characterizations, the precise mechanisms by which disease initiates and progresses have remained elusive (Figure 1A).

One area that has received specific attention is the contribution of TLRs to periodontal disease pathogenesis. In humans, TLR single nucleotide polymorphisms (SNP) associations with periodontal disease provide a mixed picture both in the context of individual TLR involvement, as well as between TLRs. In a German cohort, a TLR4 SNP correlated with chronic periodontal disease [73]. In a cohort of Chinese individuals, a TLR4 polymorphism distinguished moderate from severe periodontal disease [74]. Contrasting these studies, in a cohort of Turkish individuals it was reported that neither TLR2, nor TLR4 polymorphisms were able to distinguish periodontal disease [75]. In the context of detection of periodontal disease-associated bacteria with periodontal disease and TLRs, there is limited information. In one study, although no association with TLR4 was observed with disease, a polymorphism in CD14, which is part of the TLR4-LPS recognition complex, was associated with periodontal disease. Further, carriage of *P. gingivalis* in individuals with advancing alveolar bone loss was associated with the TLR4 SNP [76]. In addition to TLR2 and TLR4, other TLR associations to periodontal disease have been investigated. In a Czech cohort, a TLR9 haplotype associated with disease [77], and in a recent study it was reported from a Finnish cohort that a TLR9 polymorphism may provide protection from alveolar bone loss in the absence of *P. gingivalis* [76].

In addition to clinical approaches, experimental animal modeling including non-human primates [78,79], and rodents [80,81,82,83] support that TLRs are important in the development of periodontal disease. For instance, oral delivery of *P. gingivalis* drives oral bone loss in a TLR dependent manner, whereby mice deficient in TLR2 failed to develop oral bone loss in response to *P. gingivalis* challenge [84,85], whereas mice deficient in TLR4 remained susceptible to *P. gingivalis* induced bone loss [84]. The contribution of TLR4 in periodontal disease appears to be complex, as it was reported in a separate study that TLR4 participates in *P. gingivalis*-elicited oral bone loss [86]. An important study by Burns et al. [69] extends our understanding of TLR utilization into TLR-signaling in the context of *P. gingivalis* infection, revealing that cytokine production in response to infection is dependent on TLR2, but independent of MyD88, whereas clearance of bacteria occurs independent of TLR2, yet requires MyD88.

From the microbial perspective, capacity to elicit oral bone loss is attributed to virulence factors (i.e., PAMPs), expressed by bacteria such as *P. gingivalis*, which engage these TLRs. For example, gene mutational approaches have identified that the major fimbriae [87] of *P. gingivalis* is an important factor driving TLR-dependent tissue destruction. To this end, TLR2, but not TLR4 was shown to recognize *P. gingivalis* major and minor fimbria [88], and the complex structures of *P. gingivalis* LPS have been documented to have TLR4 agonist, antagonist, and non-stimulating properties [89,90]. Thus, although the precise mechanism by which *P. gingivalis* elicits oral bone loss is not fully understood, TLRs appear to be centrally positioned as host sensors engaged by bacteria such as *P. gingivalis* and driving periodontal disease pathogenesis. To complicate matters, several groups including ours have demonstrated that cells of the periodontium, including not only canonical immune cells, but also epithelial and endothelial cells, express innate immune receptors including TLRs, thus produce immune and anti-microbial factors in response to TLR ligation [34,35,36,37,38,91,92,93]. Yet, the independent and cooperative effects of the innate sensing capacity of these cells in the context of disease have not be fully elucidated. 

It is important to emphasize that in this review, we use *P. gingivalis* as a model organism to illustrate key facets of oral connections to systemic diseases and potential linkage of these connections to TLRs and the pathways they evoke. Indeed, periodontal disease is a complex multi-factorial disease that is influenced by the composition of the subgingival microbiome and nature of the host inflammatory response, elicited by these organisms [94]. Bacteria such as *P. gingivalis* have the capacity to modulate its environment in several ways, with recent information supporting that this organism has the capacity to drive microbial dysbiosis [46], and modify host inflammatory response (immune dysregulation) through inactivation of several pro-inflammatory cytokines [95]. Although there is compelling information that has come to light surrounding *P. gingivalis*, it is important to note that *P. gingivalis* is not the only organism that has shown the capacity to evoke host immune responses locally in the oral cavity via TLRs or have the capacity to impact systemic disease progression. Indeed, organisms such as *Fusobacterium nucleatum* and others have demonstrated capacity drive inflammation in a manner that involves TLR signaling [96,97,98]. In addition to single organisms, others have shown that polymicrobial challenge evoke strong oral bone loss activities [99], and can negatively influence systemic diseases such as atherosclerosis [100]. Thus, in our attempt to focus the review, it is important to note that other oral organisms may display important attributes that lead to similar outcomes as those that we highlight herein for *P. gingivalis*.

## 4. Atherosclerosis and Involvement of TLRs

As introduced above, several systemic diseases possess a complex innate immune involvement in their pathogenesis. Cardiovascular disease is the most common cause of death in industrial countries. In 2014 cardiovascular disease accounted for 23.4% of deaths in the US [101]. Initially described as a lipid storage disease, it is now established that atherosclerotic cardiovascular disease is a complex chronic inflammatory condition, and that inflammation impacts all phases of disease from endothelial activation to vascular plaque rupture [102]. Endothelial dysfunction is a very early event associated with developing vascular disease [103]. As atherosclerosis progresses, infiltrating macrophages accumulate lipids and take on a characteristic foam cells phenotype in the blood vessel wall [7,104]. These inflammatory cells, along with an array of innate and adaptive immune cells [103,105], coalesce to form a fatty streak and establish an atherosclerotic plaque. Late stage atherosclerotic plaques are characterized by a necrotic core, that upon rupture stimulates luminal clot formation leading to complete vessel occlusion and downstream tissue ischemia [106,107].

Several groups of PRRs play central roles in the development and progression of atherosclerosis (for recent reviews please see: Sharma et al. 2016 [108] and Chistiakov et al. 2017 [109]). The early events that occur in the development of atherosclerosis generate a complex inflammatory milieu of cytokines, chemokines, and inflammatory mediators [110,111,112]. Scavenger receptors, the first group of PRRs defined (including scavenger receptor A; SR-A), CD36, and LOX-1, are used by cells such as macrophages and endothelial cells to accumulate oxidized and acetylated low-density lipoproteins [113,114]. For example, excessive intracellular accumulation of lipid by scavenger receptors transitions macrophages to the atherosclerosis-characterizing lipid-laden foam cell phenotype [115]. Animals modeling has confirmed that both SR-A and CD36 are essential in reducing atherosclerotic lesion complexity in hyperlipidemic mice [116]. In the absence of sufficient cellular cycling of intercellular lipids from these cells via ABC-transporters such as ABCA1, and ABCG1 [117,118], macrophages accumulate lipids and lesion complexity continues to evolve.

In addition to scavenger receptors, TLR involvement has been linked to various stages of atherosclerotic cardiovascular disease. In a Russian cohort, a TLR1 polymorphism was associated with increased risk of coronary artery disease [119]. In a multi-ethnic population with systemic lupus erythematosus, a TLR2 polymorphism was linked to increased risk of arterial thrombosis [120]. Similarly, it was reported that a TLR4 polymorphism associated with lower risk of carotid atherosclerosis [121]. Contrary to this finding, Zee et al. [122] reported no association between TLR4 and atherothrombosis based on the same polymorphisms studied above. Thus, as is the case with many TLR polymorphism studies, including those in the context of infectious diseases such as periodontal disease [73,74,75,76,77], there is complexity in interpreting TLR associations in the context of cardiovascular disease. Cell-based, and animal modeling studies, have mechanistically strengthened the understanding of TLR involvement in the pathogenesis of atherosclerosis. For instance, it was observed that endothelial cell expression of TLRs is elevated at sites of atherosclerosis [123], and human coronary endothelial cells cultured in the presence of a disturbed fluid flow (a characteristic of atherosclerosis), respond more robustly to TLR2 ligands [124]. Macrophage TLR expression is also modulated in disease [125,126], whereby TLR ligation provides an additional signal to induce macrophage lipoprotein uptake, promoting foam cell formation [127]. This latter element is important as this may provide linkage between innate immune activation via TLRs and regulation of scavenger receptors—the PRRs involved in lipid uptake by macrophages. TLRs also participate in other facets of CVD including promoting severity of disease [128], and has been linked to arterial remodeling [129]. Although TLRs are linked to expression of matrix metalloproteinases [130], a group of enzymes identified in the degradation the fibrous cap of vulnerable atherosclerotic plaques [131], and TLRs such as TLR4 have been detected in ruptured atheroma [132], there is no information to support that TLR ligation influences vascular plaque rupture. Thus, TLRs are poised at a variety of levels in the vasculature tissue to recognize endogenous and exogenous ligands that may facilitate the development of atherosclerosis [133]. In addition to scavenger receptors and TLRs, other PRRs have been linked to the pathogenesis of atherosclerosis. RIG-I activation has been linked to endothelial dysfunction [134]. Activation of NOD1 was shown to accelerate atherosclerosis in ApoE^−/−^ mice [135]. Interestingly, NOD2 is linked to acceleration of atherosclerosis and oral bone loss [136], as well as vascular inflammation and formation of necrotic lipid-rich atheroma in LDLR^−/−^ mice [137]. Thus, it is clear that PRRs play key roles in many facets of atherosclerosis and its progression.

Despite the array of factors commonly associated with increased risk of atherosclerosis including diet, genetics, environmental factors, and others, these alone have been estimated to account for not more than 50% of total known burden [138]. Thus, additional unknown, or poorly understood factors must contribute substantively to the total atherosclerotic burden. Within this area of poorly understood risk, one area that has been explored is infection [139,140,141]. Among the earliest literature, reports linking infection to cardiovascular disease exist. Hektoen identified an association of tuberculus meningitis with cardiovascular disease [142]. More recently, several viral and bacterial infections including periodontal disease has been associated with increased risk of cardiovascular diseases [143,144,145,146]. However, treatment-based studies in humans using antibiotics have collectively failed to provide clear evidence of treatment for infection providing benefit to cardiovascular disease endpoints [147]. Despite these negative findings, in several of these studies, regardless of outcome, the authors noted that confounding factors such as choice of antibiotic [148], cohort, and other factors including resolution of primary infection [149] may complicate interpretation of these findings. 

One area that has received warranted attention and sustained support is the connection between periodontal diseases and risk of atherosclerotic cardiovascular disease. Poor oral health is anecdotally associated with the overall poor health prognoses of patients [150]. Indeed, this idea has gained strength as, in the absence of conclusive findings, there is an epidemiologically-based literature supporting periodontal disease associations with an array of complex human conditions including cardiovascular disease [151,152,153,154], type 2 diabetes [155,156], metabolic syndrome [157], oral cancer [158], pre-term birth [159], and rheumatoid arthritis [160]. We will focus on mechanistic associations between periodontal disease and metabolic diseases including atherosclerosis, and type 2 diabetes.

## 5. Periodontal Disease and Atherosclerosis

The connection between periodontal disease and atherosclerosis is based on clinical and experimental findings (Figure 1B). Clinical findings are mixed, with studies both supportive [144,151,161], and non-supportive of associations [162,163]. A causal association between periodontal disease and atherosclerosis has not been made, and as such has complicated interpretation of this association. Regardless, *P. gingivalis* and other oral bacteria are frequently detected in [164], and have been cultured from [165], human atherosclerotic plaques. Thus, at this time it is more appropriate to consider periodontal disease as a risk factor that may contribute to the overall disease burden, rather than in the context of a causal relationship with cardiovascular disease.

Transient bacteremia with oral bacteria occurs follows activities such as routine dental treatment, or routine dental hygiene [166,167]. This route of entry to the vasculature may serve as a conduit of direct bacterial access to endothelial cells. Alternatively, oral bacteria may gain access to the vessel wall indirectly, as a consequence of host immune cell trafficking intracellular bacteria to developing atherosclerotic plaque sites (i.e., Trojan horse) [168], or as a consequence of the persistent inflammation in the oral cavity impacting the vascular compartment [169]. At the cellular level, these associations are more clearly understood. *P. gingivalis* can invade human vascular cells including human umbilical vein endothelial cells [170], and coronary arterial cells [171], and elicit an array of cytokines and cell adhesion molecules characteristic of the activated endothelium observed in atherosclerosis [172,173]. In addition, in the presence of a high lipid environment, Qi et al. [174] reported that macrophages cultured with *P. gingivalis* readily took on a foam cell phenotype, a phenotype confirmed by others including by our group [175,176]. How this might occur is poorly understood, but our recent studies suggest that macrophage foam cell response to *P. gingivalis* is partially mediated by TLR signaling [177], and possibly nuclear transcription element liver x receptor (LXR) [178], although this latter connection has not been made in the context of foam cell formation. This is relevant as LXR activation is implicated in lipid transport diseases such as Tangier’s disease [179]. Whether this system is impacted by specific TLRs in the context of *P. gingivalis* infection remains unknown. 

Animal modeling has provided unique insight into the plausibility that infection with periodontal disease-associated bacteria such as *P. gingivalis* [180,181,182,183,184], and others [185], either alone or in combination [186], may serve as a poorly understood risk factor in atherosclerosis. Initial studies by Li et al. [180] identified that chronic injection of *P. gingivalis* into the vasculature of hyperlipidemic heterozygous ApoE^+/−^ mice led to a significant enhancement of atherosclerotic plaque accumulation as determined by lipid staining of the aortic tree. This finding was extended to include oral infection, when studies by Lalla et al. [181] and Gibson et al. [182] identified that ApoE^−/−^ mice orally challenged with *P. gingivalis* possessed more vascular plaque than those not infected. Importance of pathogen virulence as a central factor driving the observed accelerated atherosclerotic phenotype has been established as similar mice orally challenge with a mutant strain of *P. gingivalis* lacking its major fimbria (a structure important in bacterial attachment to host cells [170], and oral bone loss [87]) failed to enhance plaque accumulation [182]. The process of *P. gingivalis*-elicited acceleration of atherosclerosis appears to occur rapidly as evidence of enhanced plaque accumulation occurs as early as 3-weeks following first exposure to *P. gingivalis* [187]. The observation of *P. gingivalis* accelerated-atherosclerosis does not appear to be a caveat of murine modeling as *P. gingivalis* challenge of rabbit [183], and porcine [184] models, leads to enhanced vascular plaque. From the perspective of treatment to prevent these secondary complications, antimicrobial chemotherapy using doxycycline aborted the capacity of *P. gingivalis* to evoke atherosclerosis when the bacteria were delivered intravascularly [188]. Furthermore, immunization of ApoE^−/−^ mice with a preparation of killed *P. gingivalis* prior to homologous live bacteria oral challenge protected mice from accelerated atherosclerosis [182,187]. Thus, it remains plausible that key bacteria and their virulence factors associated with periodontal disease contribute to an important and poorly understood risk of cardiovascular disease.

## 6. TLRs and *P. gingivalis*-Elicited Atherosclerosis

Studies aimed to understand how oral bacteria might accelerate atherosclerosis are emerging, and these support interesting roles for TLRs in this capacity. As noted above, in the absence of infection, TLRs contribute to several important aspects of atherosclerosis, as well as to guide elements of *P. gingivalis* infection, and oral bone loss. To this end, TLR involvement in various aspects of cell activation associated with facets of atherosclerosis development have been reported. For example, sensitization of vascular endothelium by microbial ligands such as LPS has been observed. In the context of aortic endothelial cells, it was observed that the MCP-1 response to low level LPS or *Staphylococcus aureus* lipoteichoic acid (SaLTA) exposure is enhanced when these cells encounter invasive *P. gingivalis* prior to ligand challenge [189]. This is important as the chemokine MCP-1 serves as a monocyte/macrophage recruitment factor, and the presence of increase macrophages within the vessel wall is hallmark of early development of atherosclerosis [104]. Thus, as the presence of oral bacteria in the circulation is common, the findings by Yamoto et al. [189] implicate bacteria such as *P. gingivalis* in the generation of a sensitized vascular endothelium that is poised to respond in an exaggerated manner to an array of molecules and bacteria via sensing by TLRs on the vascular surface. Indeed, LPS from *P. gingivalis* was shown to activate vascular endothelium in a TLR2-dependent manner, and that upon activation these cells supported enhanced monocyte binding [190]. Employing vascular endothelial cells, it was also reported that the addition of LPS from *P. gingivalis* may also serve to antagonize TLR4 on these cells, while concomitantly inducing the formation of multi-receptor complex that includes TLR2/1/CD36 in lipid rafts to serve as a trigger to TLR2-induced inflammatory responses [191]. How *P. gingivalis* specifically enhances monocyte localization to the vasculature is not known; however, activation of TLR2, and possibly TLR4, with subsequent enhanced integrin expression, and lipid raft formation, may be important to the early stages of cell localization to inflammatory hot-spots in the vasculature [192].

Histologic evaluation of vascular tissue in experimental animals identified enhanced TLR2 and TLR4 expression in aortic arch tissue of infected mice compared with uninfected supporting a potential involvement of TLRs in *P. gingivalis* accelerated atherosclerosis [182]. These observations have led to studies targeting TLRs in this capacity. Employing hyperlipidemic ApoE^−/−^ mice and ApoE^−/−^ mice lacking TLR2, it was observed that those lacking TLR2 failed to accelerate atherosclerosis in response to *P. gingivalis* [193], supporting that TLR2 is necessary for the infection-accelerated atherosclerosis phenotype. Interestingly, when similar ApoE^−/−^ mice deficient in TLR4 where orally challenged with *P. gingivalis*, these animals presented with exacerbated vascular plaque accumulation as compared with ApoE^−/−^ mice possessing intact TLR4, suggesting that TLR4 may play a regulatory role in this capacity [194]. From these data, TLRs possess multi-functioning roles that impact atheroma development and possibly regulation of atheroma progression. Thus, based on current data, the multi-factorial roles of TLRs make their independent and cooperative contributions to bacterial infection-accelerated atherosclerosis difficult to discern. Further research into this area is necessary to clarify these knowledge gaps.

## 7. Metabolic Disease and Periodontal Disease

Association between periodontal disease and obesity, atherosclerosis, insulin resistance, and T2D is well accepted [144,151,154,155,161,195,196,197,198,199,200,201]. Chronic inflammation is a common theme among these metabolic diseases and is shared by periodontal disease [202,203]. In obese individuals, inflammatory adipose tissue serves as a chronic aggravating factor for metabolic syndrome where adipocytes secrete adipokines that encourage macrophage infiltration [204,205,206,207], and release inflammatory cytokines such as TNF-α and IL-6. PRRs have been linked to metabolic disease. For example, NOD1 and NOD2 activation by peptidoglycan was shown to stimulate pro-inflammatory signaling and insulin resistance by adipocytes and muscle cells [208,209]. In addition to PAMPs [4], DAMP-mediated activation of NLRP inflammasome by self-derived molecules such as thioredoxin-interacting protein in response to glucose link endogenous oxidative stress to insulin resistance [210,211], while other DAMPs such as amylin (IAPP) activate NLRP inflammasome and IL-1 production in a ROS-dependent manner that does not require thioredoxin-interacting protein [212,213]. Thus, infection-associated PAMPs and host-derived DAMPs serve as inflammatory stimuli that impact metabolic disease associated inflammation. Elevated inflammation directly impairs triglyceride accumulation in adipocytes by increasing lipolysis, thus causing increase of circulatory free fatty acids (FFAs) [206]. This is problematic as the principle consequence of elevated FFAs in circulation leads to the ectopic deposition of lipids in muscle and liver, which triggers whole body insulin resistance [206,214,215,216], the hallmark of T2D. Interestingly, elevated levels of FAAs, and triglycerides have been reported in obese [217] and non-obese [218,219] individuals with periodontal disease.

An adipose tissue-microorganism connection in the development of metabolic disease has been established by various studies, thus providing evidence that adipocytes could be targets for infectious microorganisms, and thus promoting adipose inflammation [220,221,222,223]. For example, ob/ob and db/db murine models of obesity and T2D were more vulnerable to *Listeria monocytogenies* infection than controls [224]. In additional studies, it was demonstrated that *Mycobacterium ulcerans* was capable of eliciting adipocyte necrosis and apoptosis [225], and *Chlamydia pneumoniae* was capable of driving adipocyte TNF-α production [226]. These interactions are likely in part regulated by TLR sensing, as TLR ligands such as *E. coli* LPS were shown to stimulate adipocytes as well as induce their release of FFA [227]. Indeed, several groups have established that TLRs are expressed by human and mouse adipocytes [228,229,230,231]. Since TLRs recognize *P. gingivalis* and its antigens, and mediate subsequent inflammatory responses [88,191,232,233,234,235], it is plausible that adipocytes respond robustly to *P. gingivalis* or its antigens and represent a major cellular conveyor of inflammation. 

**Figure 2:**
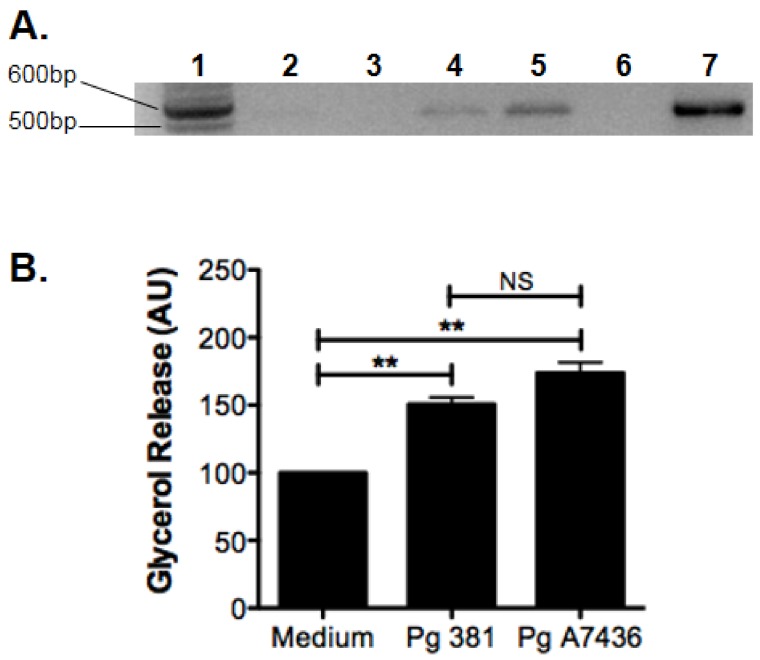
*Porphyromonas gingivalis* DNA is detected in adipose tissue excised from mice following oral challenge with live bacteria, and this organism can influence adipocyte function by enhancing glycerol release in vitro. (**A**) PCR-based detection of *P. gingivalis* 16S DNA (527 bp) in epididymal fat from unchallenged (*n* = 2) C57BL-6 mice, or 24 h following oral challenge with *P. ginigvalis* strain 381 (*n* = 2); lane 1 = bp standards, lane 2–3 = unchallenged mice, lane 4–5 = oral challenge with *P. gingivalis* 381, lane 6 = no template control, lane 7 = purified *P. gingivlais* 381 DNA as PCR control; Primers used to amplify DNA are listed in [239]. (**B**) Glycerol release (arbitrary units; AU) from murine 3T3-L1 cells cultured in RPMI-1640 medium (medium = 100%), or with medium containing killed *P. gingivalis* (Pg) strains 381 or A7436 at MOI 100 after 24 h; *n* = 3, mean+/-SEM, ANOVA with Tukey’s post-test, ** = *p* < 0.01.

To this end, similar to those in cardiovascular disease, there are experimental data to support mechanistic associations between periodontal disease and risk of metabolic disease. For instance, Endo et al. [236] reported using a ligature model of periodontal disease in rats stimulated TNF-α, IL-6, and C-reactive protein gene expression in liver and adipose tissue. In addition, in a diet-induced obesity (DIO) mouse model, mice orally challenged with *P. gingivalis* presented with reduced levels of inflammatory mediators TNF-α, IL-6, and serum amyloid A (SAA) [237]. Interestingly, exercise to reduce obesity associated risk, coupled with dietary controls was sufficient to control the inflammatory response of DIO mice to *P. gingivalis* challenge, and is accompanied by reduction in circulating TNF-α and FFA [238]. In addition, in animals, reduction of FFA levels in diet-induce obese mice challenged with the periodontal pathogen, *P. gingivalis*, restores host immune response [238]. Together, these studies suggest that *P. gingivalis* travels to adipose tissue, to elicit its effects on fatty acid metabolism. Indeed, our recent studies support this position, as we detected *P. gingivalis* in adipose tissue of orally challenged mice (Figure 2A). Furthermore, using 3T3-L1 cell line, we found that *P. gingivalis* directly derives adipocyte dysfunction by increasing lipolysis (Figure 2B).

The above studies support further detailed investigations to determine if *P. gingivalis* or other oral bacteria exacerbate adipose inflammation in obesity via TLRs on adipocytes and increase inflammatory profile of adipose tissue macrophages that culminates in increased circulatory FFAs and whole-body insulin resistance (Figure 1C).

## 8. Diabetes Mellitus and Periodontal Disease

Diabetes mellitus exists in two forms, and affects over 21 million Americans, including >9% of the adult population [240], and the classification of diabetes is based upon the pathophysiology of each form of the disease [241]. Type I diabetes is cellular-mediated auto-immune destruction of the insulin producing β-cells of the pancreas resulting in life-long dependence on exogenous insulin [241]. T2D manifests as insulin resistance whereby utilization of endogenously produced insulin is altered at the target cells [241,242]. Both forms are metabolic diseases that lead to abnormal fat, sugar, and protein metabolism. The resultant hyperglycemia ultimately induces diverse system pathologies. Secondary to hyperglycemia are several chronic sequelae including periodontal disease. 

Much research has shown the close correlation between periodontal disease and T2D [243,244,245,246,247,248,249]. For instance, a meta-analysis using 3500 diabetic adults concluded that the majority of past studies found more severe periodontal disease in diabetic patients than in adults without diabetes, confirming a significant association between periodontal disease and diabetes [250]. Also, a 2-year longitudinal study demonstrated that diabetic subjects had a significantly increased risk of alveolar bone loss compared to non-diabetic individuals, with an odds ratio of 4.2. Among those patients, poorly controlled diabetics had an odds ratio of 11.4 compared to 2.2 of well controlled diabetics [249]. The strength of evidence on the relationship between diabetes and periodontal disease have led some to suggest that periodontal disease should be listed among the “classic” complications of diabetes [244]. 

Just as diabetes contributes to increased incidence and severity of periodontal disease, periodontal disease can have a significant impact on the metabolic state of diabetics [242]. For instance, in a 2-year longitudinal trial, diabetic subjects with severe periodontitis at baseline had a six-fold increased risk of worsening glycemic control compared to diabetic subjects without periodontitis [251]. In addition, 82% of diabetic patients with severe periodontitis experienced the onset of one or more diabetic complications such as major cardiovascular, cerebrovascular or peripheral vascular events compared to only 21% of diabetic subjects without periodontitis [252]. These and other studies [251,252,253,254,255,256,257,258] support the notion that the presence of periodontal disease in diabetic patients may increase insulin resistance and contribute to worsening of the diabetic state and diabetic complications. Importantly, studies have demonstrated that mechanical periodontal treatment can improve the level of metabolic control in patients with diabetes [156,259,260]. Therefore, appropriate treatment of periodontitis in diabetic individuals is imperative to long-term health.

The mechanisms responsible for the two-way relationship between diabetes and periodontal disease is still unclear; however, infection, such as that observed in periodontal disease may serve as a metabolic stressor, resulting in an increased demand for insulin, glucose and lipids [261,262,263], as well as the systemic challenge of pyrogenic cytokines such as IL-1β, TNF-α and IL-6 [264,265,266,267]. TNF-α and IL-1β promote glycogenolyisis and impaired uptake by cells in the periphery [265,268,269,270,271,272]. In addition, they induce insulin resistance by inhibiting the insulin receptor tyrosine kinase and other signaling proteins, further increasing the physiological demand for insulin secretion [266,273,274,275]. In the context of diabetes, a hyperglycemic environment negatively influences neutrophil activities including chemotaxis [276], and bacterial killing [277]. Therefore, infectious challenge can induce or worsen a metabolic diabetic state. Since periodontitis consists of inflammatory components, which result in the production of a similar cytokine profile, this chronic disease process, could serve as a stimulus to a systemic-based inflammatory response resulting in a metabolic stressor in diabetic patients. Thus, periodontitis may enhance insulin resistance and impair insulin secretion leading to increased morbidity associated with diabetic complications (Figure 1C). 

An abnormal host immune response, or non-resolving inflammation can result in exacerbated tissue destruction. Such an abnormal inflammatory response, known as a monocytic TLR-induced hyper-inflammatory trait, has been linked to diabetes and some forms of periodontal disease [278,279]. Here, there is an increased susceptibility to infection, which is more inflammatory in nature, associated with an exaggerated secretion of innate inflammatory mediators and systemic markers of inflammation, implicating this process in the pathology associated with both chronic disease processes which may in part explain their concomitant exacerbation of each disease process. Our group has observed that oral epithelial cells derived from individuals with T2D are hyper-responsive to TLR ligation, a trait commonly observed using monocytes derived from individuals with type 1 diabetes [37,280,281]. The exacerbated TLR-induced inflammatory response from these oral epithelial cells is concomitant with an absence of TLR-induced regulatory or homeostatic responsiveness. Specifically, significantly higher concentrations of IL-8 are induced in response to *P. gingivalis* and *E. coli* LPS in epithelial cells derived from individuals with diabetes mellitus. This is relevant as IL-8 stimulates metabolism of reactive oxygen species (ROS), and promotes chemotaxis and retention of immune cells in the pancreas, all of which can amplify the inflammatory cascade [282]. Importantly, IL-8 can also induce osteoclastogenesis and osteoclast activation [283]. Thus, the overexpression of IL-8 as seen has the ability to contribute to the exacerbation of both soft- and hard-tissue destruction observed in diabetes-associated periodontal disease as well as the systemic compartments. In summary, both chronic inflammatory diseases not only share commonalities, but each disease process has a direct effect on the other, promoting a local and systemic feed-back loop resulting in exacerbation of each disease process.

## 9. Conclusions

Although the picture is complex, there has emerged an important understanding for the contribution of TLRs in the context of innate immune, and normal homeostatic function both in the context of infectious disease and chronic inflammatory diseases. This is further complicated when multiple complex chronic inflammatory diseases combine in individuals, creating a further level of complexity where TLRs may serve complementing and competing roles because of spatiotemporal events. However, as we discussed above in the context of periodontal disease associations with extra-oral diseases using connections with atherosclerosis to illustrate important overlap, and mechanistic strategies to intercede, advances in paralleling areas such as periodontal disease or other infections may provide compelling evidence regarding TLR involvement in underlying the connections between infection and systemic diseases. As periodontal disease and atherosclerotic cardiovascular disease, metabolic disease, and T2D are chronic inflammatory diseases, effectively controlling inflammation will be critical to limiting disease impact. One area that has recently shown potential benefit in the context of limiting infection-elicited inflammation and systemic disease is thru lipid mediators (for a recent comprehensive review see [284]). Among these, resolvins, represent an interesting group of lipid mediators that may provide important therapeutic potential [285,286,287,288].

Much remains to be learned and solidified regarding TLR functional involvement in microbial associated risk of extra-oral disease; however, based on current data, opportunities to intercede in disease progression by targeting TLRs may provide unique opportunity to improve not only oral health, but may also serve added benefit by reducing risk of extra-oral human health, simultaneously.
